# Differential association of ezetimibe-simvastatin combination with major adverse cardiovascular events in patients with or without diabetes: a retrospective propensity score-matched cohort study

**DOI:** 10.1038/s41598-018-30409-6

**Published:** 2018-08-09

**Authors:** Yong-ho Lee, Namki Hong, Chan Joo Lee, Sung Ha Park, Byung-Wan Lee, Bong-Soo Cha, Eun Seok Kang

**Affiliations:** 1 Division of Endocrinology and Metabolism, Department of Internal Medicine, Endocrine Research Institute, Yonsei University College of Medicine, 03722 Seoul, Republic of Korea; 2 Institute of Endocrine Research, Yonsei University College of Medicine, 03722 Seoul, Republic of Korea; 3 Graduate School, Yonsei University College of Medicine, 03722 Seoul, Republic of Korea; 4 Division of Cardiology, Department of Internal Medicine, Yonsei University College of Medicine, Seoul, Republic of Korea; 5 Cardiovascular Research Institute and Cardiovascular Genome Center, Yonsei University College of Medicine, Seoul, Republic of Korea

## Abstract

Clinical trials suggested that the benefits of ezetimibe-statin combination therapy on major adverse cardiovascular events (MACE) might be greater in patients with diabetes. We aimed to investigate the differential association of ezetimibe-statin combination with incident MACE by presence of diabetes. In this retrospective cohort study, subjects treated with simvastatin 20 mg plus ezetimibe 10 mg (S + E) or simvastatin 20 mg alone (S) between 2005 and 2015 were 1:1 matched using propensity score as stratified by diabetes. Primary outcome was newly-developed MACE composed of cardiovascular death, ACS, coronary revascularization, or non-hemorrhagic stroke. During 5,077 and 12,439 person-years, the incidence rates of MACE were 24.9, 20.1, 35.3, and 22.8/1000 person-years among no diabetes S, no diabetes S + E, diabetes S, and diabetes S + E, respectively. Relative to no diabetes S, adjusted HR (aHR) for MACE in diabetes S was 1.23 (*p* = 0.086), whereas S + E was associated with a lower risk of MACE in both non-diabetic patients (aHR 0.76, *p* = 0.047) and diabetic patients (aHR 0.60, *p* = 0.007) with significant difference (relative excess risk due to interaction = −0.39, *p* = 0.044). In conclusion, reduction of MACE risk associated with ezetimibe plus simvastatin therapy relative to simvastatin alone was greater in patients with diabetes than in patients without diabetes.

## Introduction

Atherosclerotic cardiovascular (ASCVD) disease remains the main cause of mortality and morbidity in patients with diabetes^1,2^. Despite the substantial decline in the incidence rate of diabetes-related cardiovascular complications in the past two decades, patients with diabetes still continue to have higher risk of vascular complications compared to individuals without diabetes^3^. Analyses of nationally representative data of the United States showed that patients with diabetes had 1.8- and 1.5-fold higher age-standardized rates of acute myocardial infarction (45.5 vs. 25.8 cases/10000 persons) and stroke (52.9 vs. 34.3 cases/10000 persons), respectively^3^. These findings suggest that there are still unmet needs for optimizing pharmacologic strategies to reduce cardiovascular risk in patients with diabetes.

A large randomized trial (IMPROVE-IT) including 18144 patients with recent acute coronary syndrome (ACS) showed that 10 mg ezetimibe plus 40 mg simvastatin therapy reduced the risk of major adverse cardiovascular events (MACE) compared to simvastatin alone^4^. A subsequent study from the IMPROVE-IT trial reported that a greater reduction of MACE risk was observed in patients with diabetes on ezetimibe plus simvastatin than in patients without diabetes^5^. These findings support the recommendations from recent guidelines on management of dyslipidemia that ezetimibe can be a useful non-statin therapy in high-risk patients with diabetes and established ASCVD^6–9^. However, studies focused on the differential effect of ezetimibe-statin combination therapy versus statin alone on CV outcomes among patients with or without diabetes in real-world settings are scarce yet.

In this study, we conducted a retrospective observational cohort study with propensity score matching to compare the association of ezetimibe plus simvastatin therapy versus simvastatin alone with the risk of MACE in individuals with or without diabetes.

## Materials and Methods

### Study design and population

**Figure 1:**
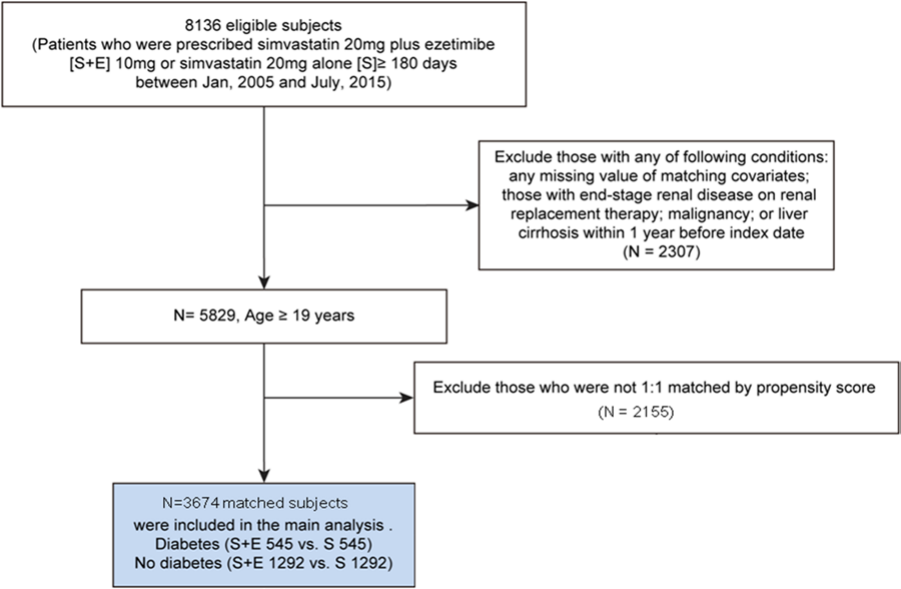
Propensity score-matched cohort study flow chart. S + E indicates individuals treated with simvastatin 20 mg plus ezetimibe 10 mg whereas S indicates those treated with simvastatin 20 mg alone. Of 8,136 subjects, 5,829 subjects without excluding criteria were first stratified by presence of diabetes. Matched S + E and S in the strata of diabetes and no diabetes remained in final analysis.

We conducted a propensity score-matched, observational, retrospective cohort study using longitudinal data retrieved from the electronic registry of the tertiary-level, university-affiliated Severance Hospital, Yonsei University College of Medicine. This study was approved by Institutional Review Board of Severance Hospital, Yonsei University (no. 4-2015-0637). All methods were performed in accordance with the relevant guidelines and regulations. Requirement to obtain informed consent was waived by the Institutional Review Board for this study based on the retrospective chart review design only with no more than minimal risk. Patients aged 19 years or older those who received prescriptions for simvastatin 20 mg plus ezetimibe 10 mg (S + E) or simvastatin 20 mg alone (S) for at least 180 days or more between January, 2005, and June, 2015 were identified to establish the study cohort. We compared the incidence of CV outcomes in 1:1 matched cohorts using propensity score (S + E vs. S) within the strata of diabetes and no diabetes and we tested the presence of significant interaction between diabetes and ezetimibe-simvastatin combination. Baseline data within 6 months before drug prescription were extracted regarding demographics, comorbidities, body mass index (BMI), smoking status, alcohol drinking status, medications, and laboratory measurements including serum total cholesterol (TC), high-density lipoprotein cholesterol (HDL-C), LDL-C, fasting glucose, and creatinine levels. Individuals with the presence of any International Classification of Diseases 10th revision (ICD-10) diagnosis codes of diabetes (E10.0–E14.9) plus evidence of continuous prescription of any type of anti-diabetes medications for ≥60 days were defined as having diabetes. The index date was the first day of S + E or S prescription. Comorbidities were identified by ICD-10 codes as follows: hypertension, I10.0, I10.1, I10.9; unstable angina, I20; myocardial infarction, I21.0-I24.9, I25.2; non-haemorrhagic stroke, I64, G46.3, G46.4, I63.9, I69.319; chronic kidney disease, N18.2, N18.3, N18.4, N18.5, N18.9. Medications at baseline were identified from the hospital prescription records using the national insurance drug codes. Blood samples after overnight fast were assessed using an auto-analyzer (Hitachi 7600-110 automated chemistry analyzer, Hitachi Company, Tokyo, Japan) at the central laboratory of the Severance Hospital. Individuals with end-stage renal disease on renal replacement therapy, malignancy, or liver cirrhosis before the index date were excluded from the analysis (Fig. 1).

### Outcomes

The primary composite outcome was MACE composed of CV death, ACS (non-fatal myocardial infarction or unstable angina requiring hospitalization), coronary revascularization (percutaneous coronary intervention or coronary artery bypass graft), and non-fatal, non-hemorrhagic stroke. The occurrence of MACE was identified by ICD-10 codes entered at the time of hospital admission or at emergency department during follow-up periods with ascertainment by diagnosis codes entered at the time of hospital discharge. Consistency of CV outcomes was adjudicated by reviewing the individual medical records. The causes of death were confirmed based on death certificates and/or primary discharge diagnosis codes at last admission. Data of subjects were followed until incident MACE, lost to follow-up, death, or 31 December 2015.

### Statistical analysis

Logistic regression models were established to estimate the propensity score for being allocated to S + E or S in each stratum of diabetes and no diabetes. For the estimation of propensity score, clinical characteristics (sex, age, BMI, current smoking, history of hypertension, unstable angina, non-fatal myocardial infarction, ischemic stroke, chronic kidney disease, current use of aspirin, clopidogrel, beta-blocker, renin-angiotensin system blockers, serum glucose, total cholesterol, LDL-C, HDL-C, triglyceride, creatinine levels, current use of metformin, sulfonylurea, insulin, and other diabetes medications) were included as model predictors. A nearest-neighbor algorithm on a 1:1 basis without replacement within a caliper of 0.01 was used to match patients. We checked covariate balance using standardized difference of mean between groups with a threshold of 10% to determine substantial imbalance (Supplemental Figs 1 and 2). The time to occurrence of MACE between matched groups was compared by plotting Kaplan-Meier curves with a log-rank test. To compare the influence of diabetes on the association between the ezetimibe-statin combination therapy and MACE, we calculated hazard ratio [HR] and adjusted HR of MACE associated with the use of S + E (vs. S) as stratified by diabetes. To test whether there is a significant interaction between the effect of ezetimibe-simvastatin combination therapy on MACE and presence of diabetes, we first calculated the relative excess risk due to interaction (RERI) as a formal test of additive interaction, which is known as the best measurement for interaction in Cox model^10^. RERI is determined as HR_11_ − HR_10_ − HR_01_ + HR_00_ in which HR_00_ denotes S users without diabetes (reference population). HR_10_ and HR_01_ represent S + E users without diabetes or S users with diabetes. HR_11_ denotes the HR of individuals with diabetes those who received S + E treatment. RERI = 0 means no interaction, whereas RERI > 0 or RERI < 0 means presence of significant positive or negative interaction. To test the presence of interaction in multiplicative scale, we next included a product term of presence of diabetes and treatment groups (S + E vs. S) in the multivariate Cox model and tested the statistical significance of coefficient of the product term. A two-sided p value of <0.05 was considered as significant. All statistical analyses were performed with STATA 12.0 (Stata Corp., TX, USA).

### Data availability

The datasets used and/or analyzed during this study are available from the corresponding author on reasonable request.

## Results

### Baseline characteristics

Among 8136 eligible subjects, we included 5829 subjects for propensity score matching after exclusion of individuals with any documented history of end-stage kidney or liver diseases and malignancy before index date. After propensity score matching, a total of 3674 subjects (S + E 545 vs. S 545 in diabetes group; S + E 1292 vs. S 1292 in no diabetes group) remained in the end for this analysis (Fig. 1). Approximately 50% of the subjects were men with a mean age of 68 years (Table 1). Most patients (81.4%) did not have any history of MACE. Baseline variables did not differ significantly among matched S + E and S groups within the strata of diabetes and no diabetes. Compared with patients without diabetes, patients with diabetes had higher prevalence of older age, male sex, previous unstable angina, non-fatal MI, more CV medications use, higher triglyceride levels, and lower HDL-C and LDL-C levels.

**Table 1:** Baseline characteristics of matched subjects according to the presence of diabetes.

	Diabetes			No diabetes			Diabetes vs. No diabetes
	S + E (n = 545)	S (n = 545)	P	S + E (N = 1292)	S (N = 1292)	P	P
Age (years)	70.3 ± 0.9	70.3 ± 0.9	0.997	67.7 ± 10.0	67.7 ± 10.9	0.985	<0.001
Men (%)	298 (54.7)	295 (54.1)	0.855	624 (48.3)	640 (49.5)	0.529	0.002
BMI (kg/m^2^)	25.1 ± 3.1	25.0 ± 3.4	0.776	24.4 ± 3.0	24.4 ± 3.1	0.681	<0.001
Current smoker	91 (16.7)	85 (15.6)	0.621	172 (13.3)	165 (12.8)	0.683	0.013
HTN	317 (58.2)	319 (58.5)	0.902	553 (42.8)	546 (42.3)	0.781	0.551
CKD	20 (3.7)	17 (3.10)	0.616	23 (1.8)	21 (1.6)	0.761	0.001
Previous UA	55 (10.1)	46 (8.4)	0.347	71 (5.5)	67 (5.2)	0.726	<0.001
Previous MI	29 (5.3)	27 (5.0)	0.784	40 (3.1)	37 (2.9)	0.729	0.001
Previous stroke	46 (8.4)	57 (10.5)	0.255	114 (8.8)	116 (9.0)	0.890	0.597
Aspirin	378 (69.4)	367 (67.3)	0.474	814 (63.0)	803 (62.2)	0.655	0.001
Clopidogrel	187 (34.3)	181 (33.2)	0.701	368 (28.5)	404 (31.3)	0.122	0.020
Beta blocker	267 (49.0)	254 (46.6)	0.431	482 (37.3)	491 (38.0)	0.715	<0.001
ACEi/ARB	311 (57.1)	295 (54.1)	0.329	551 (42.7)	539 (41.7)	0.633	<0.001
Insulin	100 (18.4)	92 (16.9)	0.525	0 (0.0)	0 (0.0)	n/a	n/a
Metformin	287 (52.7)	290 (53.2)	0.856	0 (0.0)	0 (0.0)	n/a	n/a
Sulfonylurea	234 (42.9)	226 (41.5)	0.624	0 (0.0)	0 (0.0)	n/a	n/a
Other DM med*	181 (33.2)	182 (33.4)	0.949	0 (0.0)	0 (0.0)	n/a	n/a
TC (mg/dl)	178 ± 48	179 ± 51	0.814	188 ± 50	188 ± 48	0.685	<0.001
LDL-C (mg/dl)	101 ± 40	101 ± 40	0.842	110 ± 43	111 ± 39	0.763	<0.001
HDL-C (mg/dl)	46 ± 13	46 ± 11	0.753	50 ± 12	50 ± 12	0.949	<0.001
Triglyceride (mg/dl)	163 ± 136	161 ± 109	0.773	141 ± 98	143 ± 82	0.573	<0.001
Fasting glucose (mg/dl)	130 ± 43	129 ± 39	0.716	100 ± 18	101 ± 22	0.399	<0.001
HbA_1C_ (% [mmol/mol])	7.3[56] ± 2.5	7.2[55] ± 1.3	0.364	n/a	n/a	n/a	n/a
Creatinine (mg/dl)	1.03 ± 0.45	1.01 ± 0.36	0.496	0.94 ± 0.44	0.96 ± 0.47	0.188	<0.001

Notes: Data are presented as mean ± standard deviation or number (%) as appropriate.

Abbreviations: ACEi/ARB, angiotensin-converting enzyme inhibitor or angiotensin receptor blocker; BMI, body mass index;; CKD, chronic kidney disease; DM, diabetes mellitus; HDL-C, high density lipoprotein cholesterol; HTN, hypertension; LDL-C, low density lipoprotein cholesterol; MI, myocardial infarction; S, simvastatin 20 mg alone; S + E, Combination of simvastatin 20 mg + ezetimibe 10 mg; TC, total cholesterol; UA, unstable angina.

*Composite of dipeptidyl-peptidase IV inhibitors, peroxisome proliferator-activated receptor gamma agonists, glucagon-like peptide-1 agonists, and alpha-glucosidase inhibitors.

### Outcomes

**Figure 2:**
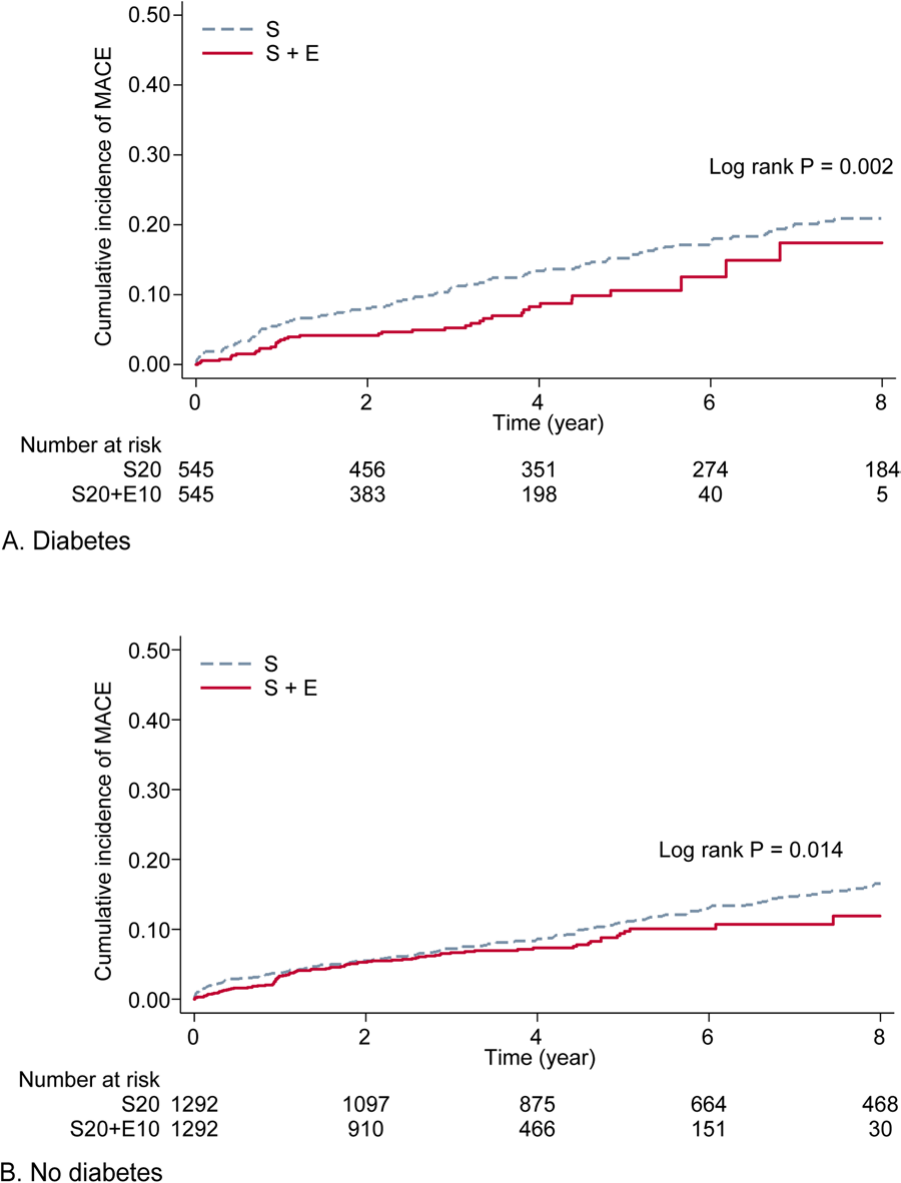
Kaplan-Meier curves for major adverse cardiovascular events (MACE) among simvastatin 20 mg alone and simvastatin 20 mg plus ezetimibe 10 mg groups stratified by the presence of diabetes. S indicates individuals treated with simvastatin 20 mg alone and S + E indicates individuals treated with simvastatin 20 mg plus ezetimibe 10 mg. The cumulative incidence of MACE in S (dash line) and S + E (solid line) group was compared (**A**) in individuals with diabetes and (**B**) those without diabetes.

During follow-up periods of 5,077 and 12,439 person-years, 157 of 1,090 in patients with diabetes and 289 of 2,584 in patients without diabetes developed MACE, respectively [median follow-up duration 4.0 (interquartile range 2.2 to 6.6) and 4.2 years (2.2 to 7.1) in diabetes and no diabetes groups, P = 0.180]. Patients with diabetes had higher incidence rates for MACE than patients without diabetes (30.9 vs. 23.2 per 1000 population per year, incidence rate ratio 1.32, 95% CI 1.08 to 1.62, P = 0.005). Among no diabetes S, no diabetes S + E, diabetes S, and diabetes S + E groups, the incidence rates of MACE were 24.9, 20.1, 35.3, and 22.8 per 1000 population per year, respectively (Table 2). Patients those who received S + E had lower cumulative incidence of MACE than those who received S within the strata of diabetes and no diabetes (Fig. 2). In Cox proportional hazard model, unadjusted HR for MACE with the use of S + E (vs. S) were 0.56 (95% CI 0.39 to 0.82, P = 0.002) and 0.72 (95% CI 0.56 to 0.94, P = 0.015) in patients with or without diabetes, respectively. The association of ezetimibe-simvastatin combination therapy with MACE remained robust after adjustment for covariates in patients with diabetes (adjusted HR 0.52, 95% CI 0.39 to 0.75, P = 0.002) and in non-diabetic patients (adjusted HR 0.76, 95% CI 0.59 to 0.98, P = 0.047). Among individual components of MACE, ezetimibe-statin combination therapy was associated with a lower incidence rate of ACS (3.7 and 8.9 per 1000 person-years, incidence rate ratio 0.41, P = 0.025) compared to simvastatin alone in patients with diabetes (Table 3), whereas lower incidence rate for CV mortality, coronary revascularization, and ischemic stroke by S + E did not reach statistical significance among diabetes and no diabetes groups. Patients with diabetes had higher incidence rate for all-cause mortality compared with those without diabetes (6.6 vs. 3.8 per 1000 person-years, incidence rate ratio 1.71, P = 0.013), whereas the incidence rate of mortality did not differ significantly between S + E and S groups within each stratum of diabetes and no diabetes. Major reasons for all-cause death were as follows: multiorgan failure due to septic shock or pneumonia (N = 31, 34.0%), solid or hematologic cancer (N = 15, 16.4%), and uncontrolled bleeding (N = 13, 14.3%).

**Table 2:** Incidence of major adverse cardiovascular event for combination of ezetimibe 10 mg plus simvastatin 20 mg compared to simvastatin 20 mg alone in subjects with or without diabetes.

	Events	Events per 1000 person-year	Unadjusted HR (95% CI)	P value	Adjusted HR (95% CI)	P value
Diabetes						
S (n = 545)	116	35.3	Reference		Reference	
S + E (n = 545)	41	22.8	0.56 (0.39–0.82)	0.002	0.52 (0.35–0.75)	0.001
No diabetes						
S (n = 1,292)	200	24.9	Reference		Reference	
S + E (n = 1,292)	89	20.1	0.72 (0.56–0.94)	0.015	0.76 (0.59–0.98)	0.047

Notes: Covariates included in the adjusted model are as follows: age, sex, history of hypertension, ischemic stroke, revascularization, use of aspirin, clopidogrel, beta-blocker, serum glucose, and serum total cholesterol.

Abbreviations: HR, hazard ratio; S, simvastatin 20 mg alone; S + E, combination of simvastatin 20 mg plus ezetimibe 10 mg.

**Table 3:** Incidence of composite and individual outcomes for combination of ezetimibe 10 mg plus simvastatin 20 mg compared to simvastatin 20 mg alone in subjects with or without diabetes.

Event number (Incidence rate, per 1,000 person-years)	S + E	S	IRR (95% CI)	P-value
All-cause mortality				
Diabetes	12 (6.3)	26 (6.7)	0.93 (0.42–1.92)	0.865
No diabetes	21 (4.4)	32 (3.5)	1.26 (0.69–2.27)	0.396
MACE				
Diabetes	41 (22.8)	116 (35.3)	0.64 (0.44–0.92)	0.013
No diabetes	89 (20.1)	200 (24.9)	0.80 (0.62–1.03)	0.087
CV mortality				
Diabetes	0 (0.0)	1 (0.3)	0.00 (0.00–79.04)	0.669
No diabetes	0 (0.0)	1 (0.1)	0.00 (0.00–75.45)	0.659
ACS				
Diabetes	7 (3.7)	33 (8.9)	0.41 (0.15–0.95)	0.025
No diabetes	17 (3.6)	50 (5.7)	0.64 (0.34–1.13)	0.107
Coronary revascularization				
Diabetes	26 (14.2)	62 (17.5)	0.81 (0.49–1.30)	0.374
No diabetes	60 (13.3)	124 (14.7)	0.90 (0.65–1.24)	0.544
Ischemic stroke				
Diabetes	8 (4.2)	20 (5.3)	0.79 (0.30–1.88)	0.602
No diabetes	12 (2.5)	25 (2.8)	0.92 (0.42–1.90)	0.834

Notes: Numbers were presented as 1,000 person-years.

Abbreviations: MACE: major adverse cardiovascular events composite of CV mortality, ACS, coronary revascularization, and ischemic stroke; CV, Cardiovascular; ACS, Acute coronary syndrome (composite of unstable angina and/or non-fatal acute myocardial infarction); IRR, incidence rate ratio.

### Interaction between diabetes and ezetimibe-simvastatin therapy

Relative to no diabetes S group, adjusted HR for diabetes S group was 1.23 (95% CI 0.97 to 1.58, P = 0.086), whereas S + E treatment was associated with a lower risk of MACE in both patients without diabetes (adjusted HR 0.76, 95% CI 0.59 to 0.98, P = 0.047) and patients with diabetes (adjusted HR 0.60, 95% CI 0.42 to 0.87, P = 0.007). Estimated RERI was −0.39 (95% CI −0.78 to −0.01, P = 0.044), which indicates a larger decrease of MACE risk by ezetimibe-simvastatin combination than expected in patients with diabetes, indicating presence of significant negative interaction between diabetes and ezetimibe-simvastatin combination therapy. Interaction for MACE in multiplicative scale was also found to be significant between presence of diabetes and ezetimibe-simvastatin combination (HR 0.64, 95% CI 0.41 to 0.98, P = 0.043).

## Discussion

In this propensity score-matched cohort study, we found that ezetimibe-simvastatin combination therapy was associated with a lower incidence rate of MACE compared with simvastatin monotherapy. The association of ezetimibe-simvastatin combination with lower risk of MACE was more enhanced in individuals with diabetes than in those without diabetes.

Our findings align with the results from a subsequent study based on the IMPROVE-IT data showing that the benefit of adding ezetimibe to statin was enhanced in patients with diabetes compared to patients without diabetes following recent ACS^5^. In this study, we found that the effect of ezetimibe-statin combination therapy on CV risk reduction was greater in patients with diabetes than in patients without diabetes and the study included broader spectrum of patients with or without previous ASCVD in real-world setting. The incidence rate of MACE observed in this study was comparable to previously published reports^4,11^. Among individual components of MACE, the incidence rate of ACS was significantly lower in S + E than in S in subjects with diabetes. In contrast, the difference of CV mortality between treatment groups did not achieve statistical significance, which was consistent with results of previous clinical trials and meta-analysis^4,12,13^. However, these findings could also be attributed to limited statistical power to detect CV mortality as a separate clinical outcome due to the low number of events observed in this study. The association of ezetimibe-simvastatin combination therapy with a lower incidence rate of MACE can be supported by previously reports. In the PRECISE-IVUS Trial, ezetimibe add-on therapy yielded greater coronary plaque regression in patients with stable angina or an ACS compared to statin monotherapy^14^. Furthermore, lifelong genetic inhibition of NPC1L1 was found to be associated with reduced risk of coronary heart disease, suggesting an essential role of NPC1L1 on protection from CV disease^15^. These findings may portend the possibility of pleiotropic effects of ezetimibe on the atherosclerosis beyond the lipid-lowering effect. We found that the association between ezetimibe-simvastatin combination therapy and all-cause mortality did not reach statistical significance in this study. This might be attributed to relatively lower number of mortality cases than expected due to exclusion of subjects with any failure in kidney or liver function at baseline and the limited identification of mortality by issued death certificate at a single institution, although further studies are needed to confirm our findings.

Several biological mechanisms have been proposed for beneficial effects of ezetimibe–statin combination therapy in individuals with diabetes. Complementary decrease in LDL-C and non-HDL-C levels by ezetimibe combination therapy was significantly greater in patients with type 2 diabetes than those without diabetes^16,17^. NPC1L1 protein expression, a direct target of ezetimibe, was enhanced by hyperglycemia in cultured intestinal cells and mRNA expression of NPC1L1 was also increased in type 2 diabetes patients^18,19^. In addition to improvement of lipid parameters, ezetimibe had favorable effects on fasting plasma glucose, insulin levels, and insulin resistance in patients with diabetes^20^. Of note, in a randomized placebo-controlled trial, 2 months of ezetimibe combination therapy did not change baseline glucose level but significantly improved insulin sensitivity, visceral fat area, and plasma adiponectin levels compared with statin monotherapy^21^. Diabetes and obesity are also known to activate the systemic inflammation^22^. In a pooled analysis of randomized, placebo-controlled trials of patients with hypercholesterolemia, the addition of ezetimibe to statin treatment significantly reduced C-reactive protein over statin monotherapy, although further studies are needed to validate this concept^23^.

Our study has several limitations. Despite the use of propensity score matching, possibilities of residual confounding due to study design and overestimation of treatment effect could not be excluded. Higher baseline CV risks of patients with diabetes might contribute to our results, although we found the presence of significant interaction between the presence of diabetes and ezetimibe-simvastatin combination therapy for the risk of MACE. Analyses on individual outcomes included in MACE in multivariate models were not possible due to insufficient number of cases for each outcome and limited statistical power. Since comparator statin was limited to simvastatin 20 mg for this study, we could not make any inference regarding combination of ezetimibe with other types of moderate to high intensity statins.

## Conclusions

Ezetimibe-simvastatin combination therapy was associated with a lower incidence rate of MACE in individuals with or without diabetes, relative to simvastatin monotherapy. The beneficial effect of ezetimibe-simvastatin combination on the risk of MACE was more pronounced in individuals with diabetes compared to those without. Considering the worldwide epidemic of diabetes, combination therapy of ezetimibe and statin might provide an effective strategy to combat residual CV risks in diabetes, which merit further investigation.

## Electronic supplementary material

Supplementary information
