# Supplementary material for: Differential association of ezetimibe-simvastatin combination with major adverse cardiovascular events in patients with or without diabetes: a retrospective propensity score-matched cohort study
Source: Sci Rep. 2018 Aug 9;8:11925. doi: 10.1038/s41598-018-30409-6 (PMC6085319; doi:10.1038/s41598-018-30409-6)
Supplement: Supplementary file 1 — Supplementary information [file 41598_2018_30409_MOESM1_ESM.pdf]

Differential association of ezetimibe-simvastatin combination with major adverse cardiovascular events in patients with or without diabetes: a retrospective propensity score-matched cohort study

Yong-ho Lee,[1,2†] Namki Hong,[1,3†], Chan Joo Lee,[4,5] Sung Ha Park,[4,5] Byung-Wan Lee,[1,2] Bong-Soo Cha,[1,2] Eun Seok Kang[1,2*]

[1]Division of Endocrinology and Metabolism, Department of Internal Medicine, Endocrine Research Institute, Yonsei University College of Medicine, Seoul, Republic of Korea, 03722.

[2]Institute of Endocrine Research, Yonsei University College of Medicine, Seoul, Republic of Korea, 03722.

[3]Graduate School, Yonsei University College of Medicine, Seoul, Republic of Korea, 03722.

[4]Division of Cardiology, Department of Internal Medicine, Yonsei University College of Medicine, Seoul, Republic of Korea

[5]Cardiovascular Research Institute and Cardiovascular Genome Center, Yonsei University College of Medicine, Seoul, Republic of Korea

†These authors contributed equally to the study.

Correspondence: Eun Seok Kang, MD, PhD

Department of Internal Medicine, Yonsei University College of Medicine,

50-1, Yonsei-ro, Seodaemun-gu, Seoul, Republic of Korea, 03722

Tel +82 2 2228 1968 ; Fax +82 2 393 6884 ; Email: edgo@yuhs.ac

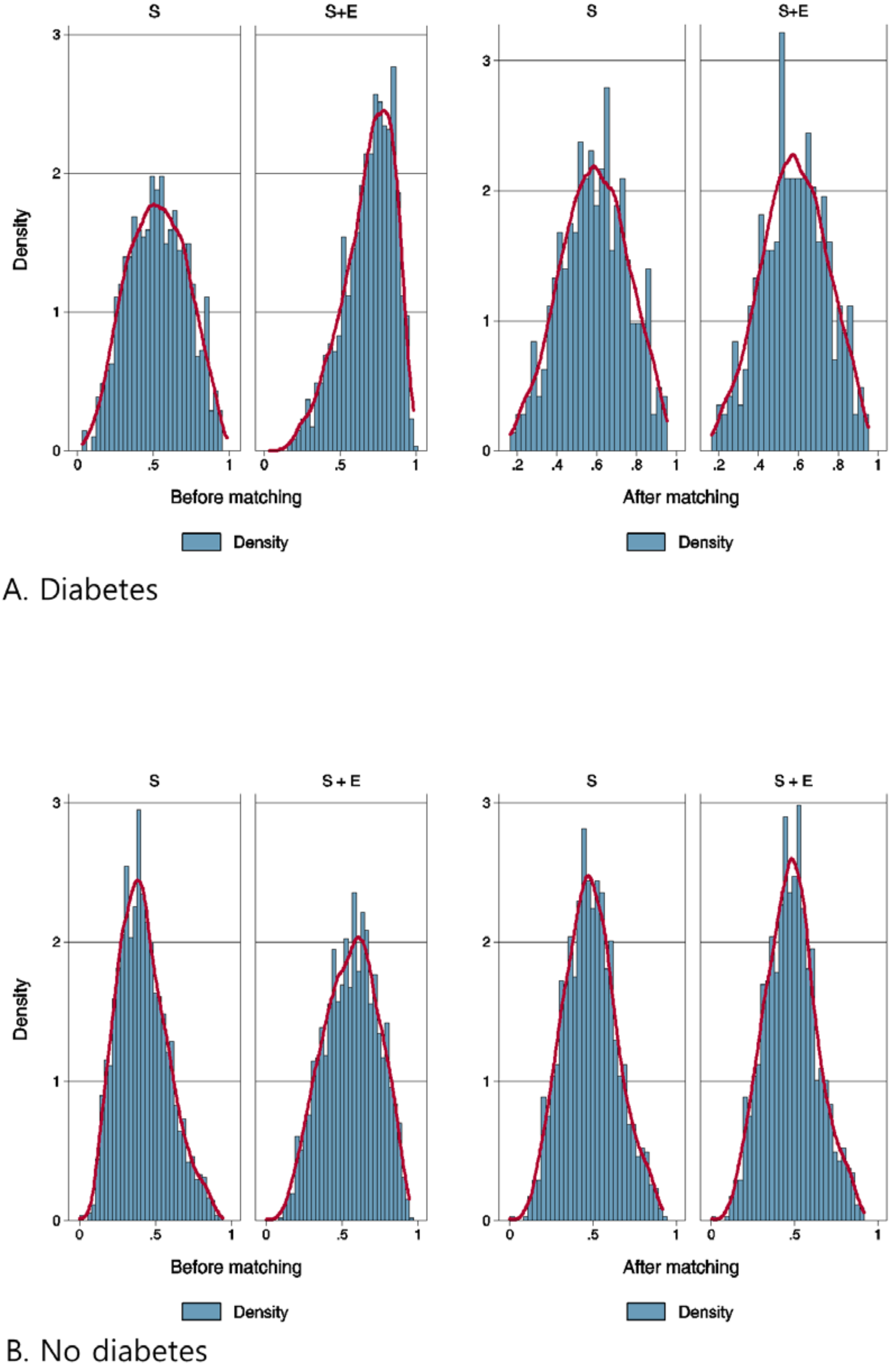

Supplemental Figure 1. Comparison of propensity score distribution before and after matching as stratified by presence of diabetes. S and S+E represent simvastatin 20mg group and simvastatin 20mg + ezetimibe 10mg group, respectively.

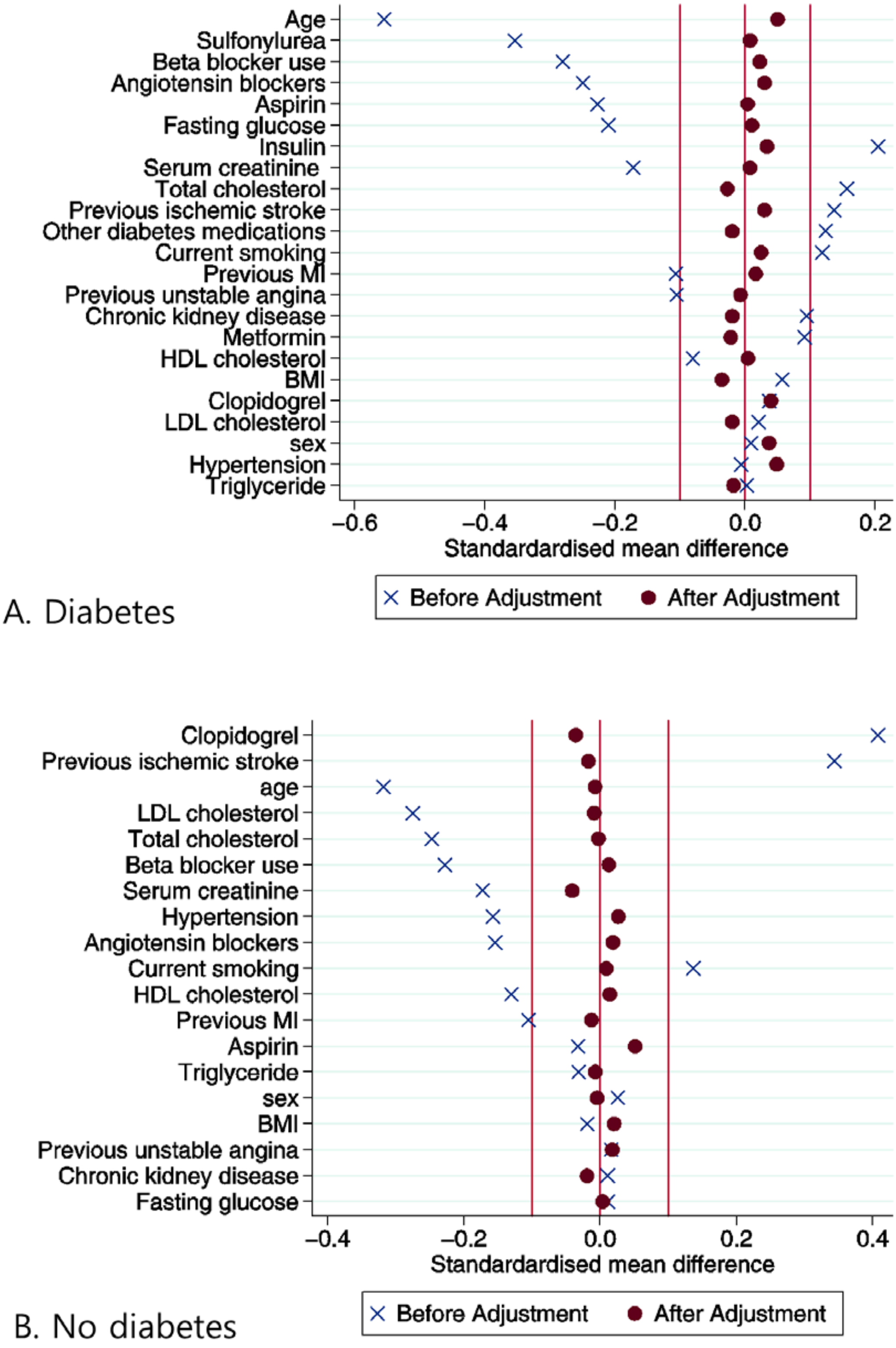

Supplemental Figure 2. Plot of absolute standardized differences for covariates before and after propensity matching between patients treated with combination of ezetimibe 10mg + simvastatin 20mg and those treated with simvastatin 20mg monotherapy (A) in patient with diabetes and (B) in patients without diabetes
